# Genetic variations of *Toll-like receptor 4* gene in exon 2 of South African Dorper sheep

**DOI:** 10.5455/javar.2024.k777

**Published:** 2024-06-08

**Authors:** Lebelo Selala, Teedzai Chitura, Vusi Mbazima, Louis Tyasi

**Affiliations:** 1 Department of Agricultural Economics and Animal Production, University of Limpopo, Polokwane, South Africa; 2 Department of Animal Science, University of Venda, Thohoyandou, South Africa; 3 Department of Biochemistry Microbiology and Biotechnology, University of Limpopo, Polokwane, South Africa

**Keywords:** DNA sequencing, genotypes, single nucleotide polymorphisms, Dorper sheep

## Abstract

**Objective:**

The study was conducted to identify the sequence variation of* Toll-like receptor 4* (*TLR4)* in exon 2 of South African Dorper sheep.

**Materials and Methods:**

Blood samples were collected from fifty (*n =* 50) South African Dorper sheep aged between 3 and 4 years. The Deoxyribonucleic acid (DNA) was extracted, amplified, and sequenced for the *TLR4* gene. DNA sequencing was used to identify the sequence variations of the *TLR4* gene in South African Dorper sheep.

**Results:**

The results showed that one synonymous single nucleotide polymorphism (SNP) of the *TLR4* gene in exon 2 position T2249C was identified. Two genotypes (TT and TC) were discovered from the identified SNP. The dominant genotype was TT (0.60) over TC (0.40), with the dominant allele T (0.80) over C (0.20). The results also indicated that the used population was in the Hady-Weinberg Equilibrium. Polymorphism genetic analysis findings suggest that the identified sequence variation of TLR4 in exon 2 of South African Dorper sheep was moderate polymorphism.

**Conclusion:**

*TLR4* gene at exon 2 of South African Dorper sheep had the SNP (T>C) at position 2249 bp with two genotypes (TT and TC).

## Introduction

The Dorper sheep was attained by crossbreeding the Dorset Horn and Black-heated Persian and has the ability to produce fast-growing lambs and the ability to survive under harsh environmental conditions [1]. This breed plays a very significant role in the lives of rural community dwellers by providing by-products such as mutton, milk, and skin and income generation [2]. However, this breed is not resistant to gastrointestinal parasites [3], hence the study related to genetic resistance is important to identify the genetic resistant sheep that might be used for breeding. *Toll-like receptors* (TLRs) are vital mechanisms of the innate immune system [4]. The* Toll-like receptor 4 (TLR4) *gene is one of the TLR and is a potential genetic marker since it is associated with disease vulnerability and resistance traits, which were conducted through single nucleotide polymorphisms (SNP) [5]. Sallam [6] indicated that this gene is found on chromosome 21. The SNPs of this gene have been studied in different sheep breeds, including Turkish sheep breeds [7] and Barki sheep [6]. Based on the knowledge of the authors, there is no documented literature on the sequence variation of *TLR4* gene in South African Dorper sheep. The work was conducted to identify the sequence variations of the *TLR4* gene in exon 2 of South African Dorper sheep.

## Materials and Methods

### Ethical approval 

The AREC/02/2020: PG was a project number for ethical approval of this study by the University of Limpopo (UL).

### Study site 

The work was done at the UL farm, which is found 9 km north-west of the university campus. The rainfall, latitude, and longitude of the area are as explained by Tyasi et al. [8].

### Experimental animals, research design, and blood collection 

The blood samples (5 ml) were collected from fifty (*n =* 50) Black-headed Dorper sheep. The blood samples were collected using vacutainer tubes containing an anticoagulant of ethylene diamine-tetraacetic acid (EDTA) (0.5%). The blood was kept at 4°C until the Deoxyribonucleic acid (DNA) was extracted [9].

### DNA extraction and amplification 

The genomic DNA was obtained from the blood samples of each animal by following Norgen’s Genomic DNA Isolation Kit protocol (Norgen Biotek Corp., Canada). A region of purified DNA was amplified by polymerase chain reaction (PCR) using forward (5’-ACCCTTGCGTACAGGTTGTTC-3’) and reverse (5’ ATGGCTGCCTAAATGTCTCAGG-3’) primers specific for a region of exon 2 of *TLR4 *[10].

### DNA sequencing 

PCR products of a region of exon 2 of the *TLR4* gene were used for sequencing to detect SNPs in the targeted region.

### Statistical analysis 

The data were analyzed using POPGENE software (version 1.32, University of Alberta, Canada).

## Results

### Amplified nucleotide sequence analysis 

**Figure 1.**
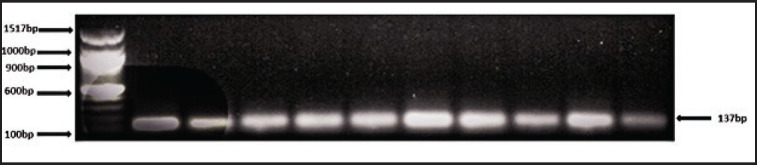
*TLR4* fragments amplicon. M, DL 1517 DNA marker (1,000bp, 900bp, 600bp, and 100bp, respectively).

Amplification was done during the PCR to detect the size of the gene. Clearly, amplified products of *TLR4* with an amplicon size of 137 bp are shown in Figure 1.

### Sequencing analysis of TLR4 gene in Dorper sheep

**Figure 2.**
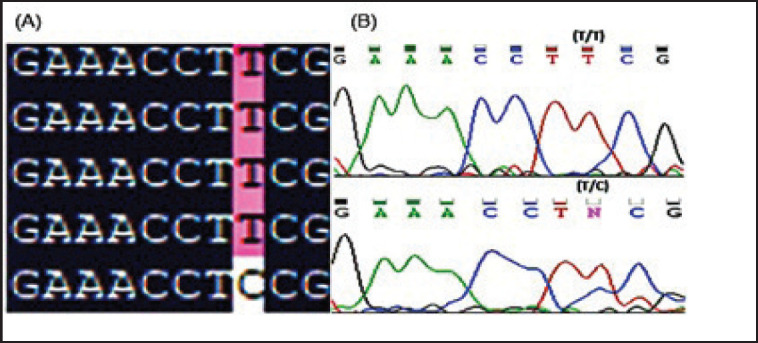
Sequence analysis (A) DNAMAN analysis and (B) chromatograms analysis. Nucleotide sequence analysis showing T2249C transition in exon 2 of *TLR4* in Dorper sheep.

DNAMAN and chromatograms were used for sequence analysis. The SNP of *TLR4* was recognized, whereby a T>C transition of exon 2 at location 2249 was indicated by Figure 2.

### Gene sequence alignment

**Figure 3.**
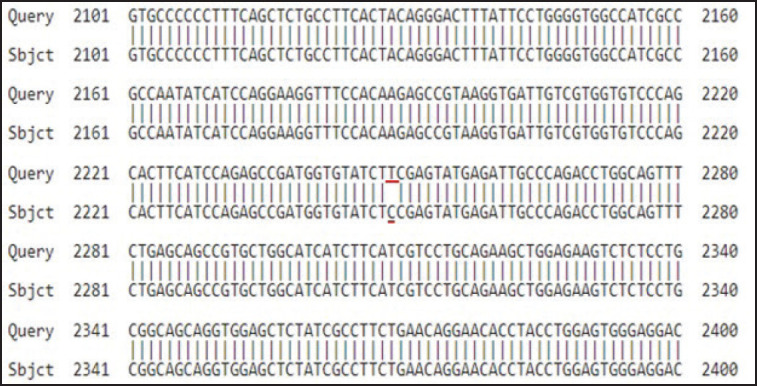
Nucleotide pairwise alignment outcome.

The blast was used to find the pairwise alignments of DNA. Figure 3 indicates DNA pairwise alignment results of *TLR4* sequence which demonstrates the location of *TLR4* SNP. The sequence alignment results showed that T2249C was in the SNP position.

### Protein sequence alignment

**Figure 4.**
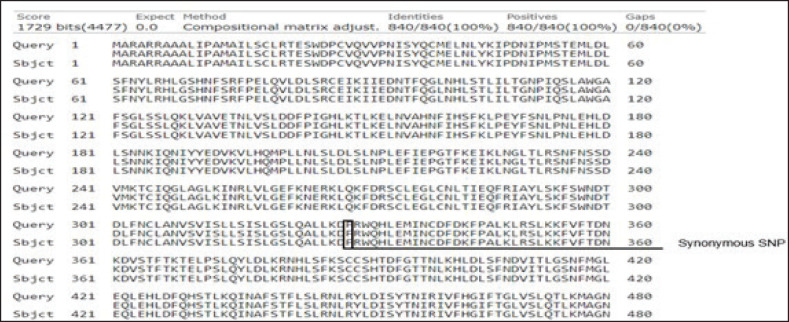
Protein sequence alignment for *TLR4 *in Dorper sheep showing synonymous SNP. Query is the protein sequence from NCBI Accession number: NP_001129402 and Subject is the protein sequence with SNP.

The blast was used to determine the sequence alignment. Figure 4 indicates the protein sequence alignment of *TLR4*. The results indicated a synonymous SNP. T>C SNP results in no change in the amino acid phenylalanine, as shown in protein sequence position 329.

### Allelic and genotypic frequencies 

The genotypic frequency of TT was higher than the genotypic frequency of TC with the allelic frequency of T being higher than the allelic frequency of C in *TLR4 *as shown in Table 1. The chi-square test showed that the examined frequencies were not significantly different from the expectations of Hardy-Weinberg.

### Population genetic analysis

The outcomes of the population genetic analysis (Table 2) showed that the heterozygosity was lower than homozygosity with an effective number (*N_e_*) of 1.47 indicating that there were moderate polymorphisms within the South African Dorper sheep population.

**Table 1.** Allelic and genotypic frequencies at the single nucleotide polymorphism locus of *TLR4* in Dorper sheep.

Genotype	*N*	Genotype frequency	Allele	Allelic frequency	*X* ^2^
TT	30	0.60	T	0.800	3.130^ns^
TC	20	0.40	C	0.200	

*p* < 0.05 was documented as statistically significant when the data were analyzed using a goodness-of-fit X^2^: chi-square test (Degree of freedom = 1).

**Table 2.** Polymorphism information analysis of *TLR4* in Dorper sheep.

SNP	Gene homozygosity (*H_o_*)	Gene heterozygosity (*H_e_*)	Effective allele number (*N_e_*)	Polymorphism information content (*PIC*)
T>C	0.680	0.320	1.471	0.269

SNP: single nucleotide polymorphism.

## Discussion

According to Astuti et al. [11], the Dorper sheep breed is one of the sheep that requires the investigation of candidate genes that might be used as genetic markers for genetic resistance. This was done to detect sequence variations in the *TLR4* gene of South African Dorper sheep using DNA sequencing. The findings of the current study revealed a nucleotide substitution of thymine (T) into cytosine (C) synonymous SNP in exon 2 of *TLR4* gene in Dorper sheep at nucleotide position 2249. DNA sequencing findings of Sallam [6] in the *TLR4* gene of Barki sheep identified a nonsynonymous mutation (rs592076818; c.1710) substitution of cytosine (C) into adenine (A) in exon 3 which results in the substitution of asparagine (Asn) into lysine (Lys) amino acids in protein sequence position 570. DNA sequencing results of Yaman [7] in *TLR4* gene of Turkish sheep breeds (4 native and 4 composite breeds) revealed fifteen SNPs and twelve of them were nonsynonymous, while three of them were synonymous on positions 873 guanine (G) change into adenine (A), 897 C change into T, and 1132 C change into T. The current study suggests that *TLR4* gene has a synonymous SNP in exon 2 that might be used as a genetic marker. The limitations of the present study are: 1) the investigated population size was small, and 2) marker-trait association was not computed. As a result of the findings of this study, more research needs to be done with a larger sample size and to conduct marker-trait associations with disease-related traits.

## Conclusion

The current study concludes that the *Toll-like receptor 4* gene in South African Dorper sheep had the sequence variation of a thymine (T) change to a cytosine (C) nucleotide at position 2249 of exon 2. Further studies need to be done on the association of the identified SNP with disease-related traits.

## List of Abbreviations

DNA, Deoxyribonucleic acid; EDTA, *Ethylenediaminete­traacetic acid*; PCR, Polymerase Chain Reaction; NCBI, National Center for Biotechnology Information; SNP, Single nucleotide polymorphism; *TLR4, Toll-like receptor 4 gene*.
